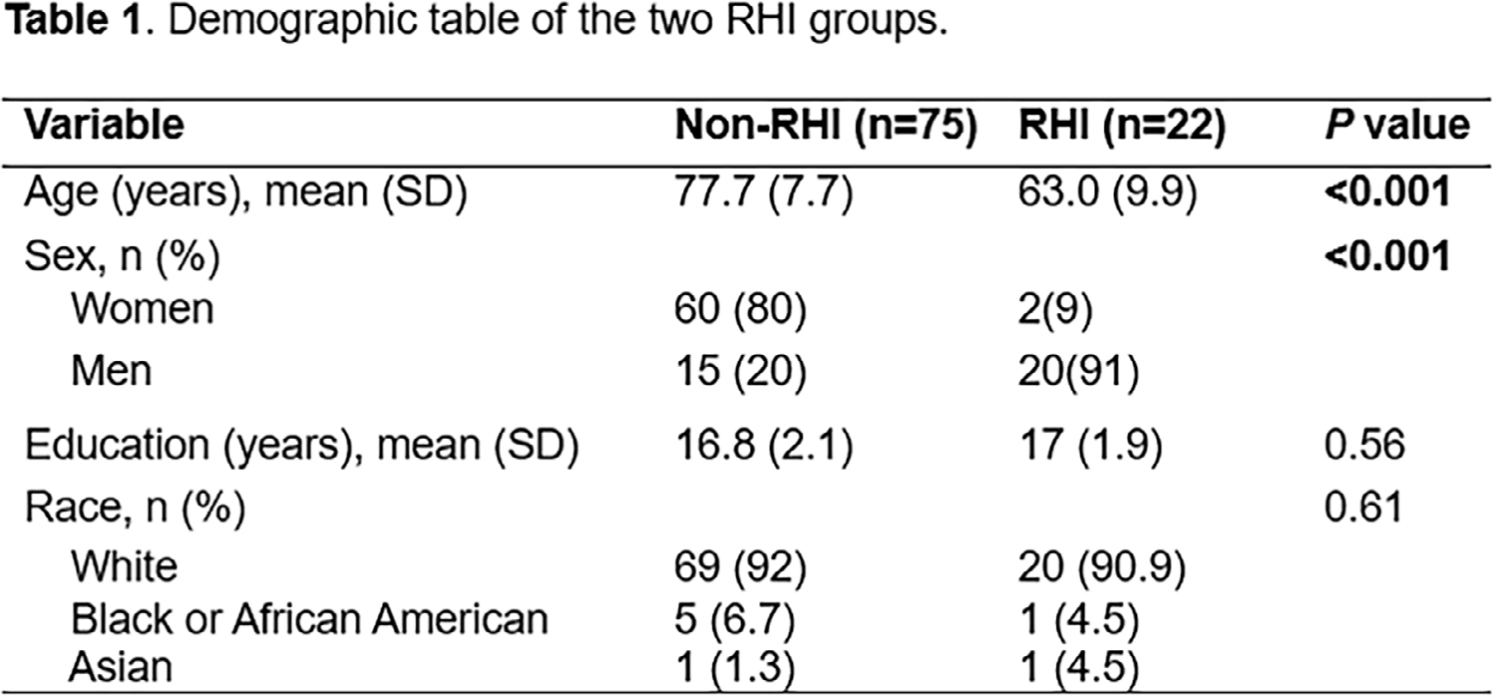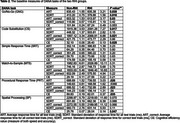# Digital Neuropsychological Measures in Older Adults Exposed to Repetitive Head Impacts

**DOI:** 10.1002/alz70856_103835

**Published:** 2025-12-26

**Authors:** Huitong Ding, Xavier Serrano, Edward Searls, Kristi Ho, Zexu Li, Alexa Burk, Margaret Low, Chenglin Lyu, Katherine A. Gifford, Vijaya B. Kolachalama, Honghuang Lin, Christopher Nowinski, Joseph N. Palmisano, Yorghos Tripodis, Katherine W. Turk, Andrew E. Budson, Ann C. McKee, Jesse Mez, Rhoda Au, Michael L. Alosco

**Affiliations:** ^1^ Boston University Chobanian & Avedisian School of Medicine, Boston, MA, USA; ^2^ University of Massachusetts Medical School, Worcester, MA, USA

## Abstract

**Background:**

Exposure to repetitive head impacts (RHI) are increasingly recognized for their association with neurodegenerative diseases. However, limited research has explored the impact of RHI on digital neuropsychological measures in cognitively intact older adults. This study aims to address this gap by comparing the cognitive performance assessed using the Defense Automated Neurobehavioral Assessment (DANA), a digital cognitive tool, between cognitively intact older adults with and without a history of RHI.

**Method:**

This study utilized data from community‐based older participants longitudinally evaluated by the Boston University Alzheimer's Disease Research Center (BU ADRC) Clinical Core for long‐term clinical outcomes associated with RHI. Participants take part in annual assessments that incorporate the Uniform Data Set. RHI classification is determined according to the 2021 National Institute of Neurological Disorders and Stroke (NINDS) Traumatic Encephalopathy Syndrome (TES) Research Diagnostic Criteria. In June 2021, participants engaged with a precision brain health monitoring digital platform that included six DANA tasks, generating five digital cognitive measures. Adjusted means and standard errors were calculated for each measure controlling for age and sex. To identify significant differences in digital measures associated with RHI exposure, Mann‐Whitney U tests were conducted on residuals of these measures adjusted for age and sex, derived from linear regression models.

**Result:**

This study included 97 cognitively intact participants from the BU ADRC (mean age: 74.4± 10.3 years; 63.9% women; mean education years: 17±2; 91.8% White) (Table 1). Of these, 22 were RHI, with football as the primary sport for 8, soccer for 5, rugby for 2, and other sports for 7. Table 2 shows the age‐ and sex‐adjusted mean values of digital measures for both groups. Participants with RHI exhibited longer average response times in the Code Substitution (2091.26 vs. 1915.36, *P* = 0.017) and Procedural Response Time (739.55 vs. 688.69, *P* = 0.026) tasks compared to those without RHI. No significant differences were observed in the Simple Response Time and Match‐to‐Sample tasks.

**Conclusion:**

Our findings indicate that RHI are associated with specific deficits in response speed among cognitively intact older adults. Results indicate subtle cognitive alterations associated with frontal mediated pathways linked to RHI exposure.